# New Method of Applying Iodine for the Cure of Phthisis Pulmonalis

**Published:** 1841-11

**Authors:** 


*New Method of Applying Iodine for the Cure of Phthisis Pulmonalis.*—The following is Mr. Leigh's method of using iodine. He directs the patient to apply a sufficient quantity of iodine ointment on the ribs, under both axillæ, and to cover the head with the bedclothes, to breathe iodine volatilized by the heat of the axillæ. The ointment produces counter-irritation on the skin where it is applied, and is to be repeated according to circumstances. This method, he says, has appeared to him to arrest the progress of phthisis.—*London Med. Gaz.*, May, 1841. *Ibid.*

